# A Success with Cocaine

**Published:** 1885-04

**Authors:** J. Smith Dodge


### A SUCCESS WITH COCAINE.

BY J. SMITH DODGE, JR., M. D., D. D. S.

Mrs. G.'s teeth have for many years been one of my extreme cases of rapid and discouraging decay, with very great sensibility. Nothing but patience and plastics could have kept any of them in her mouth. This lady came in the other day with a spot so sensitive that she could not touch it without pain, and she hardly thought I could do anything with it. It proved to be a broad, decalcified patch, caused by a clasp. The softened dentine was still in situ, and the gentlest probing was hardly tolerated. To this surface I applied, without much hope, a drop of the five per cent. solution of the oleate of cocaine. This is not an oily solution of the hydrochlorate, but a true chemical union of oleic acid with cocaine, bearing such a relation to the hydrochlorate as, for instance, the acetate of morphia does to the sulphate. The surface was gently dried, and the saliva kept at bay, while a drop was applied and allowed ten minutes to act. There was then a decided lessening of pain, but too much still remained. Another drop was applied for ten minutes, and then the entire mass of decalcified dentine was removed, and the sound dentine scraped without pain. The pulp was nowhere nearly approached. A more perfect result I cannot conceive.

